# The relation between low carbohydrate diet score and psychological disorders among Iranian adults

**DOI:** 10.1186/s12986-021-00546-3

**Published:** 2021-01-30

**Authors:** Zohreh Sadat Sangsefidi, Amin Salehi-Abarghouei, Zahra Sadat Sangsefidi, Masoud Mirzaei, Mahdieh Hosseinzadeh

**Affiliations:** 1 Nutrition and Food Security Research Center, Shahid Sadoughi University of Medical Sciences, Yazd, Iran; 2 Department of Nutrition, School of Public Health, Shahid Sadoughi University of Medical Sciences, Yazd, Iran; 3 Psychiatry and Behavioral Sciences Research Center, Ibn-e-Sina Hospital, Faculty of Medicine, Mashhad University of Medical Sciences, Mashhad, Iran; 4 Yazd Cardiovascular Research Centre, Shahid Sadoughi University of Medical Sciences, Yazd, Iran

**Keywords:** Diet, Psychological disorders, Low carbohydrate diet

## Abstract

**Background:**

Since evidence regarding to low carbohydrate diet (LCD) and psychiatric disorders is little and controversial, this study aimed to assess relation between LCD score and psychological disorders including depression, anxiety, and stress among a large representative sample of Iranian adult population in Yazd city, Iran.

**Methods:**

This cross-sectional analysis was conducted on data of 7165 persons who participated in the recruitment phase of Yazd Health Study (YaHS) and Taghzieh Mardom-e-Yazd (TAMIZ) study. Dietary intakes were evaluated by a validated semi-quantitative food frequency questionnaire. LCD score was calculated for each person according to summing up assigned scores to deciles of percentages of energy from macronutrients. Assessment of psychological disorders was also conducted by the validated Iranian version of depression, anxiety and stress scale questionnaire (DASS 21). Eventually, association between adherence to LCD and psychological disorders was evaluated via logistic regression.

**Results:**

After adjusting the confounders, women in the third quartile of LCD score might had 38% lower chance of depression versus those in the first quartile (odds ratio (OR) = 0.62, confidence interval (CI) = 0.42–0.93). However, no significant relationship was observed for other psychological disorders.

**Conclusions:**

More adherences to LCD might be associated with lower chance of depression only among women. Further studies special prospective studies are required to validate these results.

## Introduction

Depression, anxiety, and stress are among the most common psychological disorders in the world [1, 2] and are one of the important risk factors of stroke, cardiovascular disease (CVD), and some cancers [3, 4]. The prevalence of depression and anxiety has been reported as 4.4 and 3.6% in the worldwide respectively [5], whereas the prevalence rates of anxiety and depression among Iranian adults were estimated as 21.0 and 20.8% respectively [6].

Diet as a modifiable factor can affect psychological health. However, most previous studies regarding dietary factors and psychological disorders have been focused on individual macronutrients rather than their combination. For example, the results of a prospective study showed that high intakes of protein had a protective effect on depressive symptoms [7]. Low protein intakes were also associated with increased risk of psychological disorders in a cross-sectional research among Japanese male workers [8]. Furthermore, low carbohydrate along with high protein and fat consumptions were resulted in higher satiety and better mood [9]. Higher dietary glycemic index was also related to higher chance of psychiatric disorders [10, 11].

Carbohydrates are the main source of energy in the diet of Asian populations [12]. Several studies on Asian populations such as Koreans [13, 14], Japanese [15], and Chinese [16, 17] found that a high carbohydrate diet or more consumption of white rice was related to increasing risk of disease such as type 2 diabetes, metabolic syndrome and cardiovascular disease. Moreover, evidence has been shown that fat consumption among Asian populations such as Koreans and Japanese was lower than Western populations [12]. One cross-sectional study among Korean adults also indicated that a very low fat diet could increase risk of disease such as metabolic syndrome [18]. Thus, available evidence demonstrates that a high carbohydrate low fat diet may have a main role in the progression and management of disease specially in Asian countries [12].

Low carbohydrate diet (LCD) score is a newly suggested approach of macronutrient diet scores which can provide a comprehensive approach to diet–disease associations and can be more suitable to explanation of related diet and risk of chronic diseases [19, 20]. LCD considers the proportion of all dietary macronutrients in the form of a dietary pattern and defined as a diet with lower intakes of carbohydrates and higher intakes of proteins and fats [21–23].

Several studies were performed regarding relation between LCD and risk of chronic disease such metabolic syndrome [12, 24, 25], diabetes [19, 26], cardiovascular disease [20, 27], and cancer [28, 29]. Nevertheless, a few studies especially large scale studies have been evaluated association between LCD and psychological disorders and their findings were inconsistence [23, 30–33]. One research did not find any significant association between LCD score and psychological disorders (depression, anxiety and psychological distress) in a sample of Iranian adults [23], while the findings of other studies indicated a protective effect of this dietary pattern on depression in diabetic women [30] and overweight or obese women [31, 32]. More adherence to LCD was also associated with reduced chance of anxiety among diabetic women [30], overweight or obese women [32] and as well as stress in a sample of Iranian nurses [33].

Thus, regarding the important role of dietary intakes in the prevention and management of psychiatric disorders and little evidence linking LCD and these problems, the current cross-sectional study was performed to evaluate relationship between LCD score and psychological disorders including depression, anxiety and stress among a large representative sample of Iranian adult population.

## Materials and methods

### Study population and data collection

In the present cross sectional analysis, data from the recruitment phase of Yazd Health Study (YaHS) and Taghzieh Mardom-e-Yazd (TAMIZ) Study were used. YaHS is a population-based cohort study which has been conducted a large population of residents (20–69 years old) in Yazd city. Adults (n = 10,000) from 200 clusters were randomly selected from Yazd population according to residential postal codes using cluster sampling method. Yazd Nutrition Survey, locally known as TAMYZ in Persian, has assessed dietary and supplements intakes of participants of YaHS using a validated food frequency questionnaire (FFQ). More details of the mentioned studies have been published elsewhere [34]. The research was approved via the Ethics Committee of Shahid Sadoughi University of Medical Sciences, Yazd, Iran (Ethical approval code: IR.SSU.SPH.REC.1399.147, Date: September 16, 2020). Moreover, written informed consents were taken from all subjects. Information on socio-demographic characteristics, tobacco use, history of chronic disease, psychological health and physical activity assessments and dietary evaluation was obtained by a validated questionnaire. Furthermore, anthropometric assessments were conducted. In the present research, subjects with following exclusion criteria were excluded: having under or over estimation (total daily energy intake less than 800 or higher than 6500 kcal), pregnancy, following a special diet, having history of chronic disease such as CVD, diabetes, and cancer. Finally, 7165 participants were entered in the current study.

### Dietary assessment

Dietary intakes assessment was conducted using a validated FFQ consisting of 178 food items which was modified version of a previously validated 168-item FFQ. Additional 10 questions relating to the consumed Yazd-specific food items were added to the original 168-item FFQ [34, 35]. Frequency and usual amount of food items consumption were asked by participants and finally amounts of intakes were converted to grams using guidelines of household scales [36].

### Computing the LCD score

For calculation of LCD score, first the participants were classified according to decile of percentages of energy from carbohydrates, proteins, and fats. For carbohydrate consumption, subjects in the lowest decile received a score of 10, adults in second decile received a score of 9 and so on down to individuals in the highest decile received a score of 1. For consuming fat and protein, assigning the scores to deciles was reversed; so that subjects in the highest decile received a score of 10 and those in the lowest decile received a score of 1. To obtain low carbohydrate diet score, the assigned points to all macronutrients were summed up which ranged from 3 to 30 and the higher score showed more adherence to LCD dietary pattern. Finally, participants were categorized according to quartiles of LCD score.

### Psychological health assessment

Psychological health assessment was conducted via the Iranian validated short version of depression, anxiety and stress scale questionnaire (DASS 24 items) [37]. DASS 21 is a short form of the self-report depression, anxiety and stress scale questionnaire (DASS 24 items) with seven items per subscale. Responders read statements about these subscales and recorded their responses according to a 4-point Likert-type scale ranging from 0 (Did not apply to me at all) to 3 (Applied to me very much or most of the time). The scores were summed for identified items for each scale. As the DASS 21 was a short version of DASS (the Long Form has 42 items), the final score of each scale was multiplied by two. Eventually, definitions of depression, anxiety and stress were as follow respectively: having the score of ≥ 10, score of ≥ 8 and score of ≥ 15.

### Anthropometric measurements

Weight was measured by Omron BF511 portable digital scale with accuracy of 0.1 kg. Height was measured in a standing position via a tape measure on a straight wall to the nearest centimeter based on standard method. Body Mass Index (BMI) was also obtained by dividing the body weight (kg) by the square of height (m).

### Physical activity assessment

Physical activity assessment was performed by the Persian translation of short form of the International Physical Activity Questionnaire (IPAQ). Finally, physical activity level was presented as Metabolic Equivalent (MET)/min/week. Physical activity can be computed by weighting each type of activity by its energy requirements defined in the metabolic equivalent of task (MET). MET is a ratio of activity metabolic rate relative to resting metabolic rate [38, 39].

### Statistical analysis

Statistical analysis was performed using Statistical Package for Social Sciences (SPSS Corp,

version 18, Chicago, IL, USA). The normality of data was evaluated by Kolmogorov–Smirnov test. For description of data, frequency and percent or mean and standard deviation were used. Comparing characteristics of participants for categorical and continuous variables were performed by chi-square or Kruskal*–*Wallis tests respectively according to the categories of LCD score. Assessing relation between adherence to LCD with psychological disorders (depression, anxiety, and stress) among all participants and separately in men and women was conducted by logistic regression analysis in different models. In the first model, we controlled for age (20–29, 30–39, 40–49, 50–59, 60–69 years); sex (male/female); and total energy intake (Continues, kcal/day). Second model was model 1 plus additional adjustment for history of chronic disease (hypercholesterolemia, brain disease, asthma, thyroid disorders, depression, alzheimer, blood coagulation disorders, arthritis, osteoporosis; yes/no); marital status (single, married, widowed or divorced); education level (lower than high school, high school, diploma and associated diploma, bachelors, masters and Ph.D.); smoking status (never smoker, current smoker, ex-smoker); physical activity level (continues, MET/min/week); pregnancy or lactation (yes/no); dietary intakes of Eicosapentaenoic acid (EPA), Docosahexaenoic acid (DHA), and fiber (continues, g/day). Model 3 was model 2 plus additional adjustment for BMI (continues kg/m^2^). The confounding factors were chosen according to previous researches [5, 23, 30]. P for trend was also estimated by considering LCD scores as continues variables in logistic regression analysis. Furthermore, we evaluated relation between LCD score as continues variable and psychological disorders using linear regression analysis. Statistical significant level was considered as p-values less than 0.05.

## Results

### General characteristics of study population

General characteristics among all participants and according to the quartiles of LCD score have been indicated in Table 1. Among all participants, most of the subjects were aged 40–49 years (21.70%), male (50.40%), with diploma and associated diploma education (30.90%), never smoker (87.60%), and married (84.90%). Moreover, the prevalence of depression, anxiety and stress were as 8.1%, 10.5% and 3.3% respectively. The median BMI and physical activity level were 26.71 (23.50–30.02) and 719.75 (229.5–1222.68). No significant difference was observed in terms of general characteristics among participants based on the quartiles of LCD score (P˃ 0.05 for all).

**Table 1:** General characteristics of participants according to quartiles of low carbohydrate diet score

Quartiles of LCD score						
	All	Q1	Q2	Q3	Q4	*p* value
*Age (year)*						
20–29	1470 (21%)	350 (23.80%)	362 (24.60%)	391 (26.60%)	367 (25.00%)	0.96
30–39	1482 (21.20%)	353 (23.80%)	364 (24.60%)	387 (26.10%)	378 (25.50%)	
40–49	1513 (21.70%)	365 (24.10%)	356 (23.50%)	408 (27%)	384 (25.40%)	
50–59	1352 (19.40%)	329 (24.30%)	349 (25.80%)	345 (25.50%)	329 (24.30%)	
60–69	1169 (16.70%)	279 (23.90%)	291 (24.90%)	326 (27.90%)	237 (23.40%)	
*Gender*						
Male	3520 (50.40%)	863 (24.50%)	898 (25.50%)	912 (25.90%)	847 (24.10%)	0.11
Female	3465 (49.60%)	809 (23.30%)	824 (23.80%)	942 (27.20%)	890 (25.70%)	
*Education*						
Lower than high school	1697 (24.40%)	399 (23.50%)	398 (23.50%)	463 (27.30%)	437 (25.80%)	0.20
High school	1993 (28.60%)	492 (24.70%)	502 (25.20%)	533 (26.70%)	466 (23.40%)	
Diploma and associated diploma	2149 (30.90%)	522 (24.30%)	530 (24.70%)	576 (26.80%)	521 (24.20%)	
Bachelors	925 (13.30%)	210 (22.70%)	242 (26.20%)	222 (24%)	251 (27.10%)	
Masters and higher	196 (2.80%)	50 (25.50%)	35 (17.90%)	55 (28.10%)	56 (28.60%)	
*Smoking status*						
Never smoker	5976 (87.60%)	1424 (23.80%)	1453(24.30%)	1603(26.80%)	1496 (25%)	0.48
Current smoker	737 (10.80%)	179 (24.30%)	198 (26.90%)	176 (23.90%)	184 (25%)	
Ex-smoker	110 (1.60%)	29 (26.40%)	28 (25.50%)	31 (28.20%)	22 (20%)	
*Marriage status*						
Single	821 (11.80%)	192 (23.40%)	200 (24.40%)	220 (26.80%)	209 (25.50%)	0.72
Married	5904 (84.90%)	1428 (24.20%)	1451(24.60%)	1562 (26.5%)	1463 (24.8%)	
Widowed or divorced	232 (3.30%)	45 (19.40%)	57 (24.60%)	70 (30.20%)	60 (25.90%)	
*Psychological health status*						
*Depression*						0.63
Yes	549 (8.10%)	137 (25%)	141 (25.70%)	133 (24.20%)	138 (25.10%)	
No	6233 (91.90%)	1486 (23.80%)	1528(24.50%)	1664(26.70%)	1555(24.90%)	
*Anxiety*						0.79
Yes	714 (10.5%)	175 (24.50%)	178 (24.90%)	178 (24.90%)	183 (25.60%)	
No	6068 (89.50%)	1448 (23.90%)	1491(24.60%)	1619(26.70%)	1510(24.90%)	
*Stress*						0.14
Yes	221 (3.30%)	43 (19.50%)	60 (27.10%)	52 (23.50%)	66 (23.90%)	
No	6561 (96.70%)	1580 (24.10%)	1609(24.50%)	1745(26.60%)	1627(24.80%)	
*Body mass index (kg/m*^*2*^*)*	26.71 (23.50–30.02)	26.71 (23.41–29.97)	26.58 (23.50–30.11)	26.84 (23.55–30.07)	26.63 (23.52–29.90)	0.76
*Physical activity level* *(Met/min/week)*	719.75 (229.5–1222.68)	693.00 (243.25–1222.50)	700.00 (217.75–1242.50)	719.75 (236.50–1205.00)	719.75 (246.43–1200.00)	0.97

Data was presented as n (%) for categorical variables or median and interquartile range (IQR) for continues variables

Comparisons were performed using chi-square or *Kruskal*–*Wallis* tests for categorical and continues variables respectively

### Dietary intakes among participants

Table 2 shows dietary intakes among all participants and according to the quartiles of LCD score. It was found that all dietary intakes except fiber had significant differences between participants according to the quartiles of LCD score. The subjects in the higher quartile of LCD score had higher daily consumptions of energy, proteins, fats, refined grains, vegetables, legumes, dairy products, red meat, poultry, fish, eggs, nuts, EPA plus DHA than those in the lower quartile (*p* < 0.05 for all). However, individuals in the higher quartile of LCD score consumed lower amounts of carbohydrates, whole grains, and fruits versus those in the lower quartile (*p* < 0.05 for all).

**Table 2:** Dietary intakes of participants according to quartiles of low carbohydrate diet score

Quartiles of LCD score						
^*^Dietary intakes	All	Q1	Q2	Q3	Q4	^**^p value
Total energy (kcal/d)	2388.48 (1822.97–3478.91)	2379.36 (1842.89–3519.02)	2237.46 (1754.81–3390.99)	2335.54 (1817.02–3389.74)	2712.62 (1911.41–3551.04)	^**^p < 0.0001
Carbohydrates (g/d)	330.06 (252.13–473.02)	387.59 (291.65–597.21)	325.34 (255.27–499.47)	314.54 (245.25–451.10)	303.06 (217.25–398.10)	^**^p < 0.0001
Proteins (g/d)	95.31 (72.20–132.21)	79.37 (62.61–106.84)	88.26 (69.02–117.67)	97.89 (77.28–130.62)	126.34 (90.50–173.52)	^**^p < 0.0001
Fats (g/d)	82.94 (59.45–136.06)	64.45 (50.67–97.47)	73.16 (55.76–126.76)	88.98 (65.14–139.39)	115.73 (77.75–160.18)	^**^p < 0.0001
EPA plus DHA (g/d)	0.04 (0.01–0.08)	0.04 (0.01–0.08)	0.04 (0.01–0.08)	0.04 (0.01–0.09)	0.04 (0.01–0.09)	^**^p < 0.0001
Fiber (g/d)	3.14 (2.17–4.94)	3.23 (2.15–5.35)	3.11 (2.19–4.98)	3.13 (2.18–4.75)	3.13 (2.12–4.81)	0.08
Whole grains (g/d)	59.93 (26.18–92.56)	84.70 (27.79–115.89)	73.83 (27.40–95.60)	52.69 (24.02–90.66)	47.73 (25.42–87.06)	^**^p < 0.0001
Refined grains (g/d)	177.63 (113.10–288.20)	227.41 (122.21–302.35)	185.58 (108.006–295.25)	172.68 (116.35–276.19)	164.12 (101.80–274.20)	^**^p < 0.0001
Vegetables (g/d)	154.04 (104.69–251.41)	146.26 (96.09–234.71)	148.20 (104.45–234.37)	158.44 (109.83–254.73)	169.54 (106.01–269.68)	^**^p < 0.0001
Fruits (g/d)	427.33 (276.69–711.21)	507.68 (318.87–890.91)	418.26 (288.34–664.63)	410.88 (260.9–647.18)	390.09 (236.33–674.86)	^**^p < 0.0001
Legumes (g/d)	32.36 (22.08–50.85)	26.87 (19.72–41.27)	32.47 (22.61–50.69)	34.56 (23.14–52.16)	34.62 (23.83–55.50)	^**^p < 0.0001
Dairy products (g/d)	189.77 (125.34–294.88)	167.64 (109.39–261.004)	187.34 (123.21–284.64)	198.16 (131.94–303.43)	205.10 (137.24–333.76)	^**^p < 0.0001
Red meat (g/d)	38.55 (20.007–64.40)	29.60 (12.44–53.42)	34.58 (17.60–61.04)	42.86 (24.39–66.68)	61.04 (33.00–99.06)	^**^p < 0.0001
Poultry (g/d)	27.30 (15.43–74.87)	24.61 (13.65–39.17)	27.30 (14.69–51.04)	39.17 (15.43–74.78)	74.78 (27.30–145.99)	^**^p < 0.0001
Fish (g/d)	9.38 (3.45–19.34)	5.67 (2.38–13.73)	7.20 (3.45–15.21)	9.98 (5.63–22.55)	9.98 (3.65–23.62)	^**^p < 0.0001
Eggs (g/d)	17.16 (8.04–51.48)	17.16 (4.02–25.74)	17.16 (8.04–47.16)	17.16 (8.04–51.48)	25.74 (8.58–51.48)	^**^p < 0.0001
Nuts (g/d)	12.05 (6.27–23.11)	9.59 (5.44–16.02)	12.22 (6.34–23.82)	12.80 (6.82–29.95)	12.11 (6.40–27.31)	^**^p < 0.0001

^*^Data was presented as median and interquartile range (IQR). ^**^Comparisons were performed using *Kruskal–Wallis* test

### LCD score and depression

The results of assessing relation between LCD score and depression has been shown in Table 3. In first model, after adjusting the confounders including age (20–29, 30–39, 40–49, 50–59, 60–69 years); sex (male/female); and total energy intake (Continues, kcal/day), no significant association was observed between LCD score and depression in all participants (forth quartile versus first quartile: odds ratio (OR) = 0.96, confidence interval (CI) = 0.74–1.22) and men (forth quartile versus first quartile: OR = 0.85, CI = 0.58–1.24). Similarly, additional adjustments for history of chronic disease (hypercholesterolemia, asthma, thyroid disorders, depression, alzheimer, blood coagulation disorders, arthritis, osteoporosis; yes/no; yes/no); marital status (single, married, widowed or divorced); education level (lower than high school, high school, diploma and associated diploma, bachelors, masters and Ph.D.); smoking status (never smoker, current smoker, ex-smoker); physical activity level (continues, MET/min/week); dietary intakes of EPA plus DHA; fiber (all: forth quartile versus first quartile: OR = 0.98, CI = 0.75–1.28; men: forth quartile versus first quartile: OR = 0.82, CI = 0.55–1.23) and BMI (all: forth quartile versus first quartile: OR = 0.96, CI = 0.73–1.25; men: forth quartile versus first quartile: OR = 0.79, CI = 0.53–1.20) showed no significant relation between LCD score and depression in second and third models. No significant trend was also found in chance of depression across quartiles of LCD scores in all models among all subjects and men (*p* > 0.05).

**Table 3:** Multivariable-adjusted odds ratio (OR) for psychological disorders across quartiles low-carbohydrate diet (LCD) score

Quartiles of LCD score	Q1	Q2	Q3	Q4	P-trend
	OR (CI)	OR (CI)	OR (CI)	OR (CI)	
*Depression*					
*Model 1*					
All	Reference	0.98 (0.77–1.26)	0.86 (0.67–1.10)	0.96 (0.74–1.22)	0.51
Men	Reference	0.92 (0.64–1.32)	1.16 (0.82–1.64)	0.85 (0.58–1.24)	0.74
women	Reference	1.04 (0.74–1.45)	0.61 (0.42–0.88)^*^	1.04 (0.74–1.45)	0.53
*Model 2*					
All	Reference	0.96 (0.74–1.26)	0.89 (0.68–1.16)	0.98 (0.75–1.28)	0.75
Men	Reference	0.85 (0.58–1.27)	1.18 (0.82–1.71)	0.82 (0.55–1.23)	0.77
women	Reference	1.06 (0.74–1.54)	0.64 (0.43–0.94)^*^	1.10 (0.77–1.58)	0.82
*Model 3*					
All	Reference	0.95 (0.73–1.24)	0.87 (0.66–1.14)	0.96 (0.73–1.25)	0.62
Men	Reference	0.85 (0.58–1.26)	1.16 (0.80–1.68)	0.79 (0.53–1.20)	0.64
women	Reference	1.04 (0.72–1.50)	0.62 (0.42–0.93)^*^	1.08 (0.75–1.54)	0.75
*Anxiety*					
*Model 1*					
All	Reference	0.98 (0.78–1.22)	0.91 (0.73–1.13)	1.004 (0.80–1.25)	0.86
Men	Reference	0.90 (0.65–1.23)	1.14 (0.84–1.54)	0.90 (0.65–1.24)	0.96
women	Reference	1.06 (0.78–1.45)	0.69 (0.49–0.96)^*^	1.09 (0.80–1.48)	0.82
*Model 2*					
All	Reference	0.93 (0.73–1.18)	0.91 (0.71–1.15)	1.003 (0.79–1.27)	0.98
Men	Reference	0.81 (0.58–1.15)	1.11 (0.80–1.53)	0.83 (0.58–1.17)	0.70
women	Reference	1.05 (0.75–1.48)	0.71 (0.50–1.02)	1.16 (0.83–1.62)	0.77
*Model 3*					
All	Reference	0.93 (0.73–1.19)	0.91 (0.72–1.16)	1.007 (0.79–1.27)	0.98
Men	Reference	0.82 (0.58–1.16)	1.12 (0.81–1.55)	0.83 (0.58–1.18)	0.72
Women	Reference	1.04 (0.74–1.47)	0.71 (0.50–1.02)	1.16 (0.83–1.62)	0.77
*Stress*					
*Model 1*					
All	Reference	1.33 (0.89–1.98)	1.07 (0.71–1.62)	1.45 (0.99–2.18)	0.13
Men	Reference	1.36 (0.74–2.50)	1.34 (0.73–2.74)	1.40 (0.76–2.60)	0.32
Women	Reference	1.29 (0.76–2.20)	0.88 (0.50–1.54)	1.50 (0.90–2.50)	0.26
*Model 2*					
All	Reference	1.13 (0.73–1.74)	1.01 (0.65–1.56)	1.42 (0.94–2.15)	0.13
Men	Reference	1.16 (0.60–2.26)	1.36 (0.71–2.59)	1.42 (0.73–2.73)	0.24
Women	Reference	1.12 (0.63–1.98)	0.78 (0.43–1.41)	1.42 (0.83–2.45)	0.34
*Model 3*					
All	Reference	1.12 (0.72–1.72)	0.98 (0.63–1.52	1.38 (0.91–2.10)	0.18
Men	Reference	1.12 (0.58–2.18)	1.27 (0.66–2.43)	1.30 (0.67–2.52)	0.38
Women	Reference	1.11 (0.62–1.96)	0.78 (0.43–1.41)	1.42 (0.82–2.44)	0.34

CI: confidence interval

Model 1: Adjusted for sex (male/female); age (20–29, 30–39, 40–49, 50–59, 60–69); and total energy intake (continues, kcal/day)

Model 2: Model 1 + history of chronic disease (yes/no);; marital status (single, married, widow or discovered); education level (lower than high school, high school, Diploma and associated diploma, Bachelors, Masters and higher); smoking history (never smoker, current smoker, ex-smoker); physical activity level (MET/min/week); pregnancy or lactation (yes/no); intakes of dietary EPA, DHA, and fiber (continues, g/d)

Model 3: Model 2 + body mass index (BMI) (continues, kg/m^2^)

*p* < 0.05 was considered as a significance level

However, after adjusting in model 1, a significant inverse association was detected between LCD and depression in the third quartile of LCD score than the first quartile among women (OR = 0.61, CI = 0.42–0.88). This relationship did not change after additional adjusting in the models 2 (OR = 0.64, CI = 0.43–0.94) and 3 (OR = 0.62, CI = 0.42–0.93). No significant trend was also observed in odds of depression across quartiles of LCD scores in all models in women (*p* > 0.05).

Moreover, in linear regression analysis, no significant association was discovered between LCD score and depression in all participants and separately in men and women (supplementary table).

### LCD score and anxiety

Table 3 shows the findings of evaluating association between LCD score and anxiety. It was observed that there was no significant relation between LCD score and anxiety in all participants (forth quartile versus first quartile: OR = 1.007, CI = 0.79–1.27) and men (forth quartile versus first quartile: OR = 0.83, CI = 0.58–1.18) after adjustment for the confounders. No significant trend was also observed in chance of anxiety across quartiles of LCD scores in all models in all individuals and men (*p* > 0.05). Although, a significant inverse association was found between LCD and anxiety in the third quartile of LCD score in comparison to the first quartile among women after adjusting for age, sex, and total energy intake (third quartile versus first quartile: OR = 0.69, CI = 0.49–0.96), no significant relationship was observed after further adjustment for other confounders in models 2 (third quartile versus first quartile: OR = 0.71, CI = 0.50–1.02) and 3 (third quartile versus first quartile: OR = 0.71, CI = 0.50–1.02). No significant trend was also found in odds of anxiety across quartiles of LCD scores in all models in women (*p* > 0.05).

Furthermore, in linear regression analysis, no significant relationship was detected between LCD score and anxiety in all participants and separately in men and women (supplementary table).

### LCD score and stress

The results of assessing association between LCD score and stress has been presented in Table 3. According to the results, no significant relation was discovered between LCD score and stress in all participants (forth quartile versus first quartile: OR = 1.38, CI = 0.91–2.10) and separately in men (forth quartile versus first quartile: OR = 1.30, CI = 0.67–2.52) and women (forth quartile versus first quartile: OR = 1.42, CI = 0.82–2.44) after adjustment for all confounders.

No significant trend was also observed in chance of stress across quartiles of LCD scores in all models in all individuals and separately in men and women (*p* > 0.05).

In addition, in linear regression analysis, no significant association was found between LCD score and stress in all participants and separately in men and women (Additional file 1: Supplementary table).

## Discussion

This research demonstrated no significant association between LCD score and psychological disorders in all participants or men. Although, a significant inverse association was observed between LCD score and anxiety among women after adjustment for age, sex, and total energy intake, this relation was not significant after additional adjustment for other confounders. Furthermore, more adherences to LCD might be related to reduce chance of depression in women after adjusting all confounders.

Earlier studies had mostly focused on individual dietary macronutrients rather than their combination. In line with our results, one survey reported no significant association between carbohydrates or fats consumption with depression among Japanese men [8]. Furthermore, high carbohydrates consumption was associated with lower risk of anxiety and depression in obese women [40]. An inverse relation was also found between consuming high carbohydrates and high proteins with depression [41]. A few studies have been assessed relation between LCD and psychiatric disorders [23, 30–33]. Similar to our study, more adherence to LCD was related to lower risk of depression among women with type 2 diabetes [30] and overweight or obese women [31, 32]. In contrary to our findings, Ebrahimpour-Koujan et al., observed no relationship between LCD score and psychological disorders in a sample of Iranian adults [23]. Moreover, a significant association was reported between higher LCD score and decreased chance of anxiety in diabetic women [30] and overweight or obese women [32] in other studies. A protective role of LCD was also observed against stress in overweight or obese women [32] and a sample of Iranian nurses [33]. Discrepancies between others and ours might be related to differences in sample size, characteristics and health status of participants, and as well as psychological health assessment tool, and as well as computing LCD score.

Exact mechanisms regarding LCD and psychological disorders are still unknown. Nevertheless, foods containing high proteins can cause more sense of fullness and less tiredness versus foods containing high carbohydrates [42, 43]. A high refined carbohydrate diet resulted in developing depressive behaviors and anxiety in an animal study [44]. Moreover, diet rich in protein contains high amounts of amino acids including tryptophan as precursors of neurotransmitters such as serotonin that can have preventive role against psychiatric distress [33]. According to the results of Lucas et al., dietary patterns containing refined carbohydrates, sweet desserts, and sugar were related to higher risk of psychiatric disorders especially depression in women by increasing levels of inflammatory cytokines such as IL-6 and CRP [45]. In addition, It has been found that low glycemic index diet was related to decreased risk of insulin resistance and consequently reduced risk of cognitive and psychological disorders [11]. One clinical trial found that LCD had better effects on mood in compared with other restricted calorie diets after 12 months [46]. Evidence has demonstrated that LCD can inhibit glutamate decarboxylase and led to stimulating the synthesis of gamma-aminobutyric acid and eventually results in the anxiolytic/antidepressant effects [47, 48]. According to animal studies, LCD was associated with anti-inflammatory effects and increased brain-derived neurotrophic factor levels in brain that this impact might contribute to protective role against psychology disorders [48–50]. Furthermore, LCD may be effective as a mood stabilizer in depressive disorders [51]. A recent survey also suggested a possible relationship between low carbohydrate diet and depression by the mediatory role of adipokines in overweight or obese [31].

To best of our knowledge, the current research is the first population-based study regarding association between LCD and psychological disorders. FFQs were completed by a trained expert. Analyzes linking evaluation of relation between LCD and psychological disorders were also stratified by sex. Furthermore, we controlled for a wide range of confounders that might affect the psychological status of subjects. However, this study suffered from some limitations. This research cannot accurately explain the causal association among the study variables due to cross sectional nature. Although we applied valid and reliable questionnaires for evaluation of the variables such as dietary intakes, psychological health status and physical activity, some measurement bias cannot be completely avoided. In addition, we could not control the impact of all confounding factors because of unknown or unmeasured factors.

Although dietary pattern approach can provide a comprehensive insight into diet-disease associations and account complex interactions among individual foods and nutrients, the significant results in women could be from chance and they should be stated with caution due to complexity of dietary nutrition and health outcomes and as well as cross sectional design of the study.

## Conclusion

Our study did not indicate any significant relationship between LCD score and psychological disorders among all participants and men. Although, a significant inverse relation was discovered between LCD score and anxiety in women after adjustment for age, sex, and total energy intake, this association was not significant after additional adjustment for other confounders. Moreover, LCD might be associated with decreased chance of depression in women after adjusting all confounders. Further researches especially population-based longitude studies are recommended to present more conclusive evidence to clarify relation between LCD and psychological disorders.

## Supplementary Information

**Additional file 1: Supplementary Table**. Linear regression model for relation between psychological disorders and low-carbohydrate diet (LCD) score.

## Data Availability

All data generated or analyzed during this study has been included in this manuscript.

## Abbreviations

LCD: low carbohydrate diet
YaHS: Yazd Health Study
TAMIZ: Taghzieh Mardom-e-Yazd
DASS 21: short version of depression, anxiety and stress scale questionnaire
OR: odds ratio
CI: confidence interval
CVD: cardiovascular disease
FFQ: food frequency questionnaire
BMI: Body Mass Index
IPAQ: International Physical Activity Questionnaire
MET: metabolic equivalent
