# Supplementary material for: The relation between low carbohydrate diet score and psychological disorders among Iranian adults
Source: Nutr Metab (Lond). 2021 Jan 30;18:16. doi: 10.1186/s12986-021-00546-3 (PMC7847167; doi:10.1186/s12986-021-00546-3)
Supplement: Supplementary file 1 — Additional file 1: Supplementary Table. Linear regression model for relation between psychological disorders and low-carbohydrate diet (LCD) score. [file 12986_2021_546_MOESM1_ESM.docx]

**Supplementary table. Linear regression model for relation between psychological disorders and low-carbohydrate diet (LCD) score.**

|  | **LCD score** |  |
| --- | --- | --- |
|  | **Beta coefficient** | **P-value** |
| **Depression** |  |  |
| **Model 1** |  |  |
| All | -0.01 | 0.36 |
| Men | -0.01 | 0.48 |
| women | -0.01 | 0.51 |
| **Model 2** |  |  |
| All | -0.007 | 0.59 |
| Men | -0.01 | 0.58 |
| women | -0.006 | 0.74 |
| **Model 3** |  |  |
| All | -0.009 | 0.48 |
| Men | -0.01 | 0.47 |
| women | -0.007 | 0.68 |
| **Anxiety** |  |  |
| **Model 1** |  |  |
| All | -0.003 | 0.78 |
| Men | -0.006 | 0.73 |
| women | -0.002 | 0.89 |
| **Model 2** |  |  |
| All | -0.002 | 0.86 |
| Men | -0.01 | 0.57 |
| women | 0.004 | 0.80 |
| **Model 3** |  |  |
| All | -0.001 | 0.93 |
| Men | -0.008 | 0.63 |
| Women | 0.005 | 0.77 |
| **Stress** |  |  |
| **Model 1** |  |  |
| All | 0.01 | 0.33 |
| Men | 0.009 | 0.58 |
| Women | 0.01 | 0.46 |
| **Model 2** |  |  |
| All | 0.01 | 0.28 |
| Men | 0.01 | 0.45 |
| Women | 0.01 | 0.50 |
| **Model 3** |  |  |
| All | 0.01 | 0.36 |
| Men | 0.009 | 0.60 |
| Women | 0.01 | 0.51 |

Model 1: Adjusted for sex (male/female); age (20-29, 30-39, 40-49, 50-59, 60-69); and total energy intake (continues, kcal/day).

Model 2: Model 1+ history of chronic disease (yes/no);; marital status (single, married, widow or discovered); education level (lower than high school, high school, Diploma and associated diploma, Bachelors, Masters and higher); smoking history (never smoker, current smoker, ex-smoker); physical activity level (MET/min/week); pregnancy or lactation (yes/no); intakes of dietary EPA, DHA, and fiber (continues, g/d).

Model 3: Model 2+ body mass index (BMI) (continues, kg/m^2^)

P< 0.05 was considered as a significance level.
